# May-Thurner Syndrome: ﻿A Neglected Cause of Unilateral Leg Swelling

**DOI:** 10.2147/OAEM.S246018

**Published:** 2020-05-01

**Authors:** Ehab Badawy, Mohammed A Seif, Amr Elmoheen

**Affiliations:** 1 Emergency Department, Hamad Medical Corporation, Doha, Qatar

**Keywords:** May-Thurner syndrome, deep vein thrombosis, leg swelling

## Abstract

May-Thurner syndrome (MTS) is a clinical condition characterized by the compression of the left iliac vein by the right iliac artery. This condition predisposes the patient to deep venous thrombosis (DVT). We present the case of a 30-year﻿-old female who arrived at the emergency department of our facility with progressive left leg swelling for four weeks, with low-risk probability for DVT. Examination revealed left leg swelling with pitting edema extending up to the knee. Her calf muscle was tender to palpation. Dorsalis pedis, anterior tibial, and posterior tibial pulsations were fairly palpable due to the edema; however, the rest of her pulsations were appropriately felt. Therefore, the provisional diagnosis of possible DVT was made, and further investigations were requested. We present this case intending to highlight the clinical presentation of May-Thurner syndrome, its diagnosis, and treatment.

## Background

May-Thurner syndrome (MTS) is a clinical condition characterized by compression of the left iliac vein between the right iliac artery that overlies it, and the lumbar spine. May-Thurner syndrome has a prevalence rate of 22–24%.^1^ MTS presents as edema and pain in the left lower extremity. Because MTS may present as a deep venous thrombosis, effective management is essential as it may trigger a pulmonary embolism, resulting in morbidity and mortality.

Many reports suggested that the incidence of this condition is not fully known, but ranges from 18% to 49% among patients with DVT of the left-sided lower extremity.^2^

Most times, the disease goes unrecognized, with a prevalence rate three times higher in women than in men.^3^ It presents mostly between 20–40 years of age.

Standard treatment techniques include stent placement, thrombolysis, and prolonged anticoagulation. Long-term anticoagulation is mostly applied in patients who have received a stent. There have been rare cases of DVT recurrence even after venous stenting, which may call for venous bypass surgery.^3^

Summarily, the exact incidence and prevalence of MTS are unknown as most of those cases are asymptomatic and require no treatment unless there is a significant compression that causes a leg swelling, as in this case.^1^,^4^,^5^ This is a rare cause that deserves to be listed in the differential diagnosis of unilateral leg swelling.

## Case Presentation

A 30﻿-year-old-female patient presented to the emergency department with progressive painful left leg swelling for four weeks. There was no history of recent long travel, major surgeries, previous DVTs, or active malignancy. She had recurrent hospital admissions due to repeated abdominal pain and vomiting episodes. She was recently diagnosed as suspected superior mesenteric artery syndrome.

Examination revealed left leg swelling with pitting edema extending up to the knee. The left leg calf muscle was tender to palpation. The left leg dorsalis pedis, anterior tibial, and posterior tibial pulsations were fairly palpable due to the edema; however, the rest of her limbs pulsations were appropriately felt.

Therefore, the provisional diagnosis of possible DVT was made, and further investigations were requested.

## Investigations

**Figure 1:**
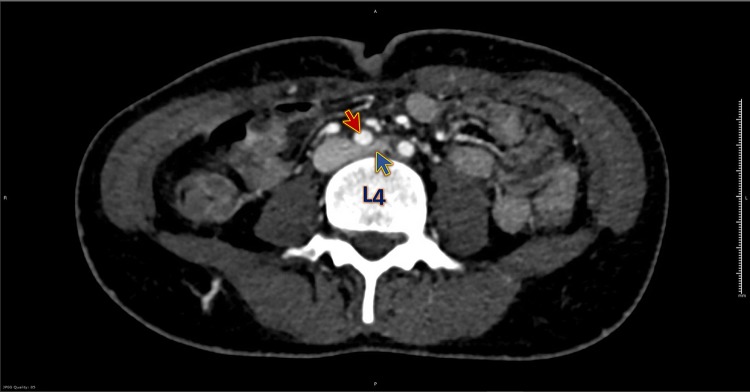
CT venogram axial view shows compression of the left iliac vein (blue arrow) by the right iliac artery (red arrow) at the level of the 4th lumbar vertebra (L4).

**Figure 2:**
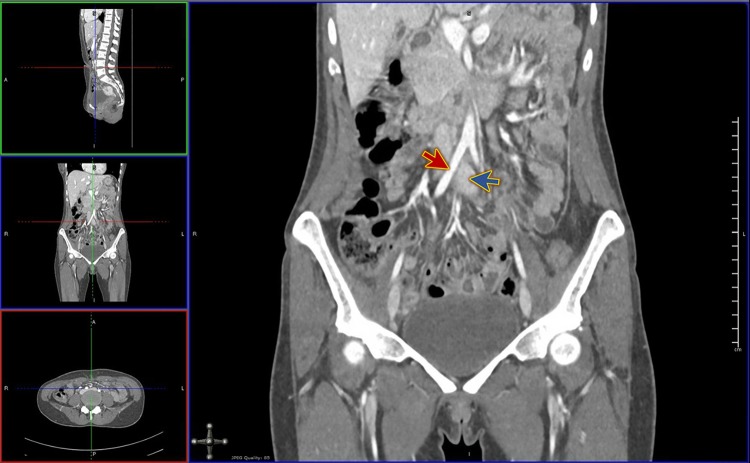
CT venogram coronal view shows compression of the left iliac vein (blue arrow) by the right iliac artery (red arrow).

**Figure 3:**
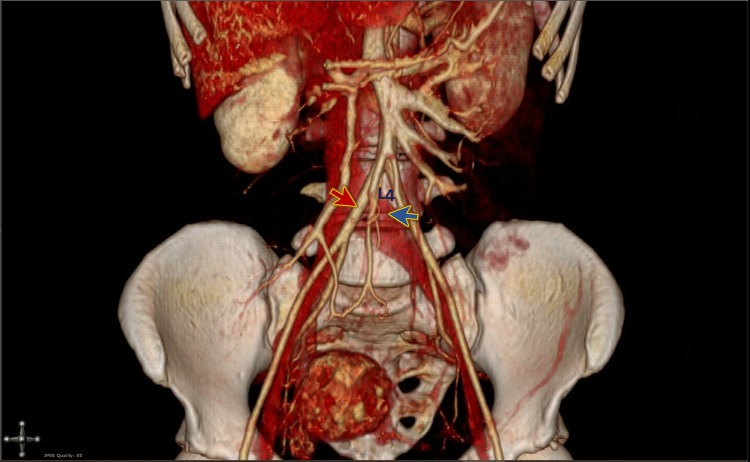
3D reconstruction of the CT venogram shows compression of the left iliac vein (blue arrow) by the right iliac artery (red arrow) at the level of the 4th lumbar vertebra (L4).

Initial blood workup came later, including a complete blood picture, renal function test, and coagulation profile, and they were normal. D-dimer was 0.38 mg/L. We did a Doppler ultrasound on the lower limb veins, which revealed no evidence of venous thrombosis. Based on the reliable clinical findings suggestive of DVT with negative Doppler US up to the femoral veins, CT venogram of the lower limb veins from the IVC was requested to look for any iliac vein thrombosis. CT venogram showed her left common iliac vein was compressed by the right common iliac artery at its origin, against the L4 vertebral body (Figures 1–3), a picture suggestive of May-Thurner syndrome.

## Differential Diagnosis

Upon presentation, we suspected deep vein thrombosis, cellulitis, and hypercoagulability. We also considered thrombophilia relative to antiphospholipid antibody syndrome (APLAS). However, the blood results showed a normal white blood cell count, normal antinuclear antibody, and anticardiolipin titers. The International Normalized Ratio (INR) was within the normal range. CT Venogram confirmed the presence of May-Thurner syndrome.

## Treatment

Due to the severity of pain and possible thrombosis, we recommended prophylactic anticoagulants.^6^ The patient was given prophylactic Dalteparin Sodium (Fragmin) 5000 International Units once daily for eight days. The patient was sent to the vascular surgery team for elective angioplasty to relieve her symptoms. She underwent placement of the iliofemoral venous stent.^7^ The patient has a smooth post-operative course. She was eventually discharged home on the tenth day on fondaparinux 2.5 mg subcutaneously once daily for 45 days.

## Outcome and Follow-Up

The patient has finished her 45 days of Fondaparinux. Her symptoms have improved dramatically. Her leg swelling has disappeared, and she has no pain in her leg. She did not develop any deep vein thrombosis after the stenting. She is still following up with the internal medicine and vascular surgery teams.

## Discussion

May-Thurner syndrome is a condition affecting 18–49% of patients with deep venous thrombosis of the left lower extremity. It constitutes a risk factor for left-sided iliofemoral deep venous thrombosis. Several postulates have been suggested concerning the pathophysiology of May-Thurner syndrome. First, its occurrence is linked to constant pulsations of the right iliac artery, which results in spur formation in the wall of the vein. This spur obstructs the region, resulting in the creation of a clot.

Secondly, because of the pulsations occurring in the overlying artery, the venous walls become traumatized, resulting in collagen and elastin deposition impairing venous return and subsequently leading to deep venous thrombosis.

Also, DVT may occur as a complication of hypercoagulability. Patients are experiencing this condition present with varicose veins, pigment changes, phlebitis, and chronic leg pain. These conditions are more prevalent in young women in their second to the fourth decade, especially after prolonged immobilization and pregnancy.^8^

Diagnosis is dependent on the clinical presentation (mostly swelling and pain in the left lower extremity). Detection of deep vein thrombosis is facilitated by Doppler ultrasound. However, it is essential to note that Doppler ultrasound cannot visualize compression of the iliac vein and spur’s location. On that note, conventional venography, Intravascular ultrasound (IVUS), helical abdominal computed tomography, magnetic resonance venography, and CT venography may also be used in diagnosis.^8^ Pelvic computed tomographic images in the transverse plane help in the detection of iliac vein compression by the common iliac artery overlying the compressed vein.^9^ Magnetic resonance imaging is an essential diagnostic tool as it helps with the detection of pelvic masses, deep venous thrombosis, and equally demonstrates the structural features of the syndrome.^10^ It is important to note that conventional venography remains the gold standard for the diagnosis of May-Thurner syndrome.^8^

MTS is treated via thrombus clearance and correction of the left iliac vein compression. The first-line treatment for May-Thurner Syndrome includes stenting and endovascular intervention with thrombolysis. The surgeon may perform catheter-directed thrombolysis involving local administration of tissue plasminogen activator (tPA-alteplase) or urokinase at the site of the thrombus. It dramatically reduces the clot burden. Mechanical thrombectomy may also be performed to minimize thrombotic infusion time or associated complications. Angioplasty with stent placement comes after thrombolysis or thrombectomy, intending to relieve the obstruction.^8^ A study by Moudgill et al involving 113 patients with left-sided deep venous thrombosis who were treated with stent placement and catheter-directed thrombolysis showed a 95% mean technical success and a 95% mean one-year patency.^3^ Hager et al did a study showing the efficacy, safety, and durability of stenting for May-Thurner syndrome, and post-thrombotic and edema patients alone. The efficacy, safety, and durability lasted for 36 months, with a 91% patency rate.^11^ If endovascular treatment fails, implantation of the stent could be done surgically. Treatment of DVT with MTS by venous thrombectomy alongside stent placement resulted in venous patency and symptom relief.^7^ Subsequently, after the clearance of thrombus and placement of the stent, patients receive routine treatment with anticoagulation for maintenance of venous patency and prevention of re-stenosis of the stent.^8^ The debate about the duration of anticoagulation comes after stent placement. Anticoagulation should be done after six months, followed by reassessment of the patient’s risk of deep venous thrombosis. This allows the physician to make an accurate decision on the duration of anticoagulation.^12^ Factors to consider at the time of reassessment include bleeding risks, patient compliance, underlying etiology of deep venous thrombosis, the presence of a venous stent, patient reference, polypharmacy, risk of recurrence, and co-morbidities.^13^

In conclusion, the highlight of this case revolves around the need to keep May-Thurner syndrome as a differential diagnosis in a 30-year old female presenting with query DVT of the lower limb. This is because uncorrected structural defects of May-Thurner syndrome can trigger a recurrence of DVT, rupture of the iliac vein, or pulmonary embolism. Another vital point to note is that systemic anticoagulation on its own is insufficient for the management of MTS cases, but works effectively as a routine treatment for deep venous thrombosis. However, extended anticoagulation remains debatable, but six months is recommended to maintain vein patency.

The patient, in our case, was referred to the vascular surgeon for possible angioplasty. Prophylactic anticoagulant was commenced until the surgery as there is a high likelihood of developing DVT. Therefore, as indicated in our case, a multidisciplinary approach is necessary for effective management of this condition.

## Learning Points/Take-Home Messages

- If there is a strong clinical suspicion of deep vein thrombosis (DVT) in the presence of a negative ultrasound doppler of the femoral veins distally, we should look for any proximal veins abnormality up to the Inferior Vena Cava.
- Broaden your differential diagnosis and keep a low threshold of suspicion of May-Thurner Syndrome, especially if the patient has no risk factors for deep vein thrombosis (DVT).
- Iliofemoral venous stent proved to have a brilliant outcome.
